# The influence of a Healthy Welcoming Environment on participation in club sport by adolescent girls: a longitudinal study

**DOI:** 10.1186/s13102-017-0076-y

**Published:** 2017-05-19

**Authors:** M. M. Casey, R. M. Eime, J. T. Harvey, N. A. Sawyer, M. J. Craike, C. M. Symons, W. R. Payne

**Affiliations:** 1 School of Health Sciences and Psychology, Faculty of Health, Federation University Australia, PO Box 663, Ballarat, VIC 3353 Australia; 2 Institute of Sport, Exercise and Active Living (ISEAL), College of Sport and Exercise Science, Victoria University, PO Box 14428, Melbourne, VIC 8001 Australia

**Keywords:** Health promotion policies, Sporting club, Adolescent

## Abstract

**Background:**

This study investigated the perceived influence of a Healthy Welcoming Environment (HWE) on participation in sports clubs among adolescent girls, and how these perceptions changed longitudinally. HWE was defined in terms of a set of health promotion policies advocated by a health promotion foundation as the basis of sport club health promotion practice to promote structural reform in state sporting organisations and their affiliated associations and clubs. These included sports injury prevention, smoke-free, responsible serving of alcohol, sun protection, healthy eating, and welcoming and inclusive environments.

**Methods:**

Year 7 and 11 female students from metropolitan (*n* = 17) and non-metropolitan secondary schools (*n* = 14) in Australia were invited to participate in three annual surveys. These surveys collected information about current or past membership of a sports club and the influence of HWEs on their decision to participate (or not) in a sports club.

**Results:**

Year 7 (*n* = 328; 74.5%) and Year 11 (*n* = 112; 25.5%) female students completed all three waves (19.6% response rate; 82.7 and 74.0% retention rate). Most agreed that characteristics of HWEs were a positive influence on their participation in sports clubs, except those relating to alcohol and Sunsmart. Welcoming factors had consistent high agreement among respondents. Alcohol and friendliness factors of the club were regarded as being positively influential by higher percentages of non-metropolitan than metropolitan respondents.

**Conclusions:**

Welcoming factors were the most positive influences on decisions to participate in sports clubs. These factors may be important in reducing barriers to sport participation. Strategies supporting the social environment within sports clubs should be prioritised.

## Background

Globally, physical inactivity is the fourth leading risk factor for mortality [1]. It is widely acknowledged that to achieve substantive health benefits, physical activity (PA) should be of at least moderate intensity, and that vigorous intensity activities can provide even greater health benefits [2]. Internationally, four-fifths of adolescents do not achieve the recommended levels of 60 min of moderate to vigorous PA per day [3]. Further, PA trends show that girls are less active than boys, and youth participation declines with age [4].

Sport settings are considered important for increasing overall PA levels, as there is potential to reach large numbers of people; particularly as sport is a type of PA which is popular among children and can contribute to overall moderate and vigorous PA [5]. Team sport participation has been associated with a reduced risk of being overweight or obese among adolescents [6], which is important since the proportion of children who are obese continues to increase [7]. In addition, club sport participation contributes to positive physical, mental and social health and has additional health benefits to other types of PA [8]. As a consequence, the sports club has been identified as a health-promoting setting, which can encourage healthier behaviours [9, 10]. In Australia, a sports club is the main setting where organised, competitive sport is played in the community.

The settings approach is a contemporary health promotion practice which has been described as signifying “the modernization of public health” (p.383) [11]. It is based on whole-systems thinking in order to introduce, manage and sustain change within a particular setting to address cultural, social, economic and environmental factors within the contexts and places that people live [12]. As such health promotion action within a sports club setting might involve introducing changes to personal health behaviours (individual level), the daily coaching practices (micro-level or intrapersonal level), club management (meso-level or organisational level) or the sport club environment (macro-level or physical environment–policy level) [13]. There is limited research on how health can be promoted through sports club settings. Research to date has mostly focused on the prevalence of health promotion policies and practices in sports clubs [14, 15] and peak sporting organisations [16, 17]; the organisational development of sporting organisations for health promotion [18, 19] and the development of standards for a health promoting sports club [20]. In Australia a set of sports club health promotion policies and practices have been defined by the Victorian Health Promotion Foundation (VicHealth) under the rubric of Healthy and Welcoming Environments (HWE) [14, 17, 21]. There were six key areas of HWE: sports injury prevention, ‘smoke-free’ environments, responsible serving of alcohol, sun protection, healthy eating, and welcoming and inclusive environments. These were aimed at promoting structural reform in state sporting organisations and their affiliated associations and clubs [14, 17, 21]. It is believed that the creation of HWEs within clubs will facilitate changes in personal health behaviours, and in particular increase club participation/membership [21]. The HWE constructs have been investigated at the organisational level in terms of policy and practice development within state sporting organisations [21, 22], however not at the individual level to understand how these health promotion practices influence sport participation. These sports club health promotion policies and practices have the potential to influence adolescents at a stage when there is a large drop-off in sport participation [23]; however this influence has not yet been examined.

A panel of Australian health and sports professionals recently identified responsible alcohol management, smoke-free environments and healthy eating as priority areas for health promotion action for community sports clubs [24]. Determining the actual influence of HWEs on sports participation is important as this knowledge may assist in the development and implementation of strategies to improve adolescent participation in sport. Such information would be particularly important to improve the participation levels of those groups, such as adolescent girls, who currently have low PA participation. The purpose of this study was to determine the perceived influence of HWEs on participation in sports clubs among adolescent girls and how these influences changed over a 3 year period.

## Methods

This study was part of a larger study which examined changes over time in PA levels and determinants of participation among adolescent girls. The methodological details of this study have been previously reported [25, 26]. Seventeen secondary schools in the metropolitan area of Melbourne, Victoria, Australia and 14 schools in surrounding rural and regional areas participated in the study.

All female students in Years 7 and 11 from participating schools were invited to participate. There were three longitudinal waves of data collection at 12-month intervals during Autumn months [25].

The survey form for the larger study included questions relating to PA levels and contexts, and potential intrapersonal, interpersonal, organisational and environmental determinants of PA [25]. The study presented here included those participants who reported being current or past members of a sports club. The participants were asked about the influence of various characteristics of HWE (listed in Tables 1 and 2) on their decision to participate or not to participate in a sports club. The key areas of the HWE practices and policies developed by VicHealth were listed (see Tables 1 and 2) and the participants were asked “Do any of the following influence you to participate or not to participate in a sports club?’ Four response options were presented: no influence, positive influence, negative influence, don’t know. Because the focus of the study was on the extent to which aspect of HWE was regarded as a positive influence, and because positive responses predominated, responses were dichotomised as ‘positive influence’ and ‘non-positive’ for analysis.

**Table 1:** Influence of characteristics of a healthy and welcoming environment: Longitudinal changes within cohorts and differences between cohorts

Characteristic	Indicator^b^	Cohort (school year level in 2008) and wave (calendar year)										Statistically significant difference between cohorts	
		Year 7 (*n* = 328^a^)					Year 11 (*n* = 112^a^)						
		2008	2009	2010	Statistically significant linear trend		2008	2009	2010	Statistically significant linear trend			
		*n* = 292^c^ 89%	*n* = 292^c^ 89%	*n* = 298^c^ 91%	*p*-value	Sign	*n* = 102^c^ 91%	*n* = 104^c^ 93%	*n* = 100^c^ 89%	*p*-value	Sign	*p*-value	Sign^d^
Healthy Environment													
The club is smoke free	% Yes	62	53	50	0.004	-	60	61	54				
The club has Sunsmart practices	% Yes	41	30	30	0.002	-	26	27	26				
The club is responsible in the serving of alcohol	% Yes	34	35	34			24	34	41	0.005	+		
The club has injury prevention strategies in place	% Yes	71	69	73			67	70	74				
Welcoming environment													
Knowing someone at a club	% Yes	87	89	90			90	91	89				
Friendliness of the coach	% Yes	80	85	87	0.045	+	86	84	90				
Skill and/or experience of the coach	% Yes	63	64	67			80	74	74			0.002	+
The day/time of competition/practice sessions	% Yes	76	74	79			67	77	77				
The club is friendly	% Yes	92	92	95			95	97	97				

^a^Sample sizes: girls who completed all three waves of the survey

^b^The indicator for each characteristic is the percentage of girls who indicated that the characteristic was positively influential

^c^Sample sizes: girls who stated that they were currently or formerly a member of a sports club in each of the three waves of the survey; percentage of all respondents

^d^Year 11 versus Year 7

**Table 2:** Influence of characteristics of a healthy and welcoming environment: Longitudinal changes within regions and differences between regions

Characteristic	Indicator^b^	Region and wave (calendar year)										Statistically significant difference between regions	
		Metropolitan (*n* = 315^a^)					Non-metropolitan (*n* = 125^a^)						
		2008	2009	2010	Statistically significant linear trend		2008	2009	2010	Statistically significant linear trend			
		*n* = 274^c^	*n* = 282^c^	*n* = 280^c^	*p*-value	Sign	*n* = 120^c^	*n* = 120^c^	*n* = 118^c^	*p*-value	Sign	*p*-value	Sign^d^
Healthy environment													
The club is smoke free	% Yes	63	54	52	0.017	-	60	57	47	0.040	-		
The club has Sunsmart practices	% Yes	38	28	28	0.006	-	35	34	31				
The club is responsible in the serving of alcohol	% Yes	31	32	33			34	41	43			0.041	+
The club has injury prevention strategies in place	% Yes	70	71	76			70	67	68				
Welcoming environment													
Knowing someone at a club	% Yes	87	90	87			90	91	95				
Friendliness of the coach	% Yes	80	83	87			84	88	89				
Skill and/or experience of the coach	% Yes	68	66	66			66	69	76				
The day/time of competition/practice sessions	% Yes	74	75	77			75	74	81				
The club is friendly	% Yes	92	92	94			97	97	100			<0.001	+

^a^Sample sizes: girls who completed all three waves of the survey

^b^The indicator for each characteristic is the percentage of girls who indicated that the characteristic was positively influential

^c^Sample sizes: girls who stated that they were currently or formerly a member of a sports club in each of the three waves of the survey

^d^Non-metropolitan versus Metropolitan

In a preliminary analysis, baseline characteristics of participants who returned survey forms in all 3 years of the study (‘completers’) and those who did not (‘non-completers’) were compared using t-tests and chi-square tests. Longitudinal analysis was based on the completers. All dependent variables analysed in this study were Yes/No dichotomies (see Tables 1 and 2). Longitudinal logistic regression fitted by the method of generalised estimating equations was used to identify statistically significant differences between the two cohorts and statistically significant longitudinal trends within each cohort. Both linear and non-linear trends were tested for, but all significant trends were linear. Differences and trends were assessed in 2-factor models incorporating cohort effects, time trends and cohort-time interactions. Because there were significant interactions, time trends were further assessed in simple effects analyses, i.e. separate analyses of trend for each cohort. All analyses were conducted using SPSS Version 21, with statistical significance set at *p* < .05.

## Results

Details of sampling design, recruitment and retention rates have been previously reported [25]. Briefly, the initial (wave 1) recruitment/response rate (the proportion of invited students who provided consent and returned the first survey form) was 19.6% with retention rates in waves 2 and 3 of 82.7 and 74.0%, respectively. Respondents who returned survey forms in all three waves of the study comprised: Year 7 (*n* = 328, 74.5% aged 11–13, M ± SD = 12.2 ± 0.5 years at baseline) and Year 11 (*n* = 112, 25.5% aged 16–18, 16.2 ± 0.6 years at baseline).

In terms of demographics and the context of sport and PA participation there were some significant differences between those who completed all three surveys (‘completers’) and those who did not (‘non-completers’). For the Year 7 cohort those who completed all three surveys (‘completers’) had a lower mean self-reported weight at baseline (M ± SD = 46.6 ± 9.3 kg; *p* = .009) than those who did not (‘non-completers’: M ± SD = 49.4 ± 10.9 kg). Across both year level cohorts, completers were significantly more likely than non-completers to report participating in PE classes at school (63.9% v 45.8%, *p* < .001), competitive team sports outside school (64.5% v 53.7%, *p* = .003), or competitive individual sports at school (68.6% v 55.1%, *p* < .001).

Responses from participants who reported being current or past members of a sports club were included in the present study. These constituted over 89% of respondents in each year level and wave (see Table 1 for details). The majority of respondents agreed that characteristics of HWEs were a positive influence on their decision to participate or not to participate in a sports club. In particular, many of the welcoming aspects (i.e. knowing someone at the club, friendliness of the coach, friendliness of the club) had high levels of agreement (>80.0%). Other welcoming aspects such as the day/time of competition/practice sessions (75.8%) and skills/experience of coach (67.9%) were also a positive influence. Health aspects were primarily reported as a positive influence (i.e. ‘Sunsmart’ sun protection practices 31.8%; responsible serving of alcohol 34.1%; smoke-free 56.0%; and injury prevention 71.0%). The second most common response for each item was “no influence”, with proportions ranging from 3.7% (club is friendly) to 55.3% (Sunsmart). Very few respondents reported “negative influence”, with less than 3.1% for each characteristic of HWEs except responsible serving of alcohol (11.0%). Further, small minorities of respondents reported “don’t know” (range: 1.5–11.4%), again with a rather higher percentage for responsible serving of alcohol (16.4%).

The longitudinal changes within ‘school year level’ cohorts and differences between cohorts regarding the percentage of respondents who reported that each characteristic positively influenced their decision of participate in a sports club is summarised in Table 1. A summary for the longitudinal changes within metropolitan and non-metropolitan regions and differences between regions is presented in Table 2.

With the exception of Sunsmart practices and responsible serving of alcohol, all other characteristics were assessed as being a positive influence by a majority of both cohorts in all three waves of the study (see Table 1). The characteristic consistently cited as a positive influence was the friendliness of the club. In the younger cohort (Year 7), a smoke-free environment and Sunsmart practices were significantly less influential over time, while the importance of the friendliness of the coach were significantly more influential over time. In the older cohort (Year 11), the only significant change over time was an increase in the percentage of respondents who regarded responsible serving of alcohol as influential. The only characteristic which exhibited a significant difference between the two cohorts was the skill and/or experience of the coach, which was reported as being influential by higher percentages of the older cohort than the younger cohort.

In terms of difference between metropolitan and non-metropolitan adolescent girls, Table 2 shows significant downward trends over time in the perceived influence of smoke-free environment in both regions, and in Sunsmart practices in the metropolitan region. The regions differed significantly with respect to responsible serving of alcohol and friendliness of the club, both of which were regarded as being positively influential by higher percentages of non-metropolitan than metropolitan respondents.

The patterns of response regarding three of the nine characteristics did not change significantly over time within cohorts or regions, or differ significantly between cohorts or regions. In descending order of the percentage of positive responses, these were: knowing someone at a club, the day/time of competition/practice sessions, and the club having injury prevention strategies in place.

## Discussion

This is the first study to examine the perceived influence of HWEs on participation in sports clubs among adolescent girls and how these influences changed over a 3 year period. This study is important to inform strategies for improving sport participation by adolescent girls. There are many determinants of participation in club sport. There is an abundance of literature focusing on the individual and social determinants but much less at the organisational or policy levels. This study focused on the health promotion policies and practices at the sport club (organisational) level that may influence adolescent participation in sport. The results identify important aspects of a sports club environment that can be developed, managed and promoted to encourage sport participation among adolescent girls.

Two of the healthy environment practices (smoke-free environments and injury prevention strategies), along with a welcoming environment, were assessed as a positive influence on sports club membership by a majority of adolescent girls in all three waves of the study. However the importance of smoke-free club environments declined over time for the younger cohort (Year 7) in both the metropolitan and non-metropolitan regions. It has been reported that alcohol and tobacco use increases as adolescents age [27], which may indicated more liberal attitudes towards these behaviours. The downward trend for the positive influence of smoke-free environments in the present study is consistent with those findings. Similarly, only around a third of respondents in the present study identified responsible serving of alcohol as important. However, responsible serving of alcohol became more important among the older cohort and within non-metropolitan areas. Research has found that risky drinking increases with remoteness [28], and specifically that adolescents within rural areas begin using alcohol at younger ages and at higher patterns of use compared to those in urban areas [29]. Further participants in sport clubs show higher levels of alcohol consumption and higher rates of risky consumption than the general community [30]. Research on alcohol management programs in sporting clubs is only emerging, although programs such as Good Sports in Australia have been linked with increased sport club membership especially among females and young people [31], as less emphasise on alcohol may broaden the appeal of being involved with a sports club [32]. It has also been suggested that alcohol management in sports clubs may be even more important in rural areas where sporting clubs are a central hub of social activity [31]. This study found that alcohol management is important in rural areas and as adolescent girls’ age. Future research could explore the impact of alcohol management on participation in sports clubs by adolescent girls.

The majority of adolescent girls in this study considered that factors associated with a welcoming environment influenced their involvement in sports clubs. More than 80.0% of adolescent girls agreed that the friendliness of the club, knowing someone at a club and friendliness of the coach were positive and influential factors affecting their sport club participation compared to other HWE practices. Previous research has identified the importance of social support in facilitating sport and PA participation, especially when activities involve being with friends [33, 34]. This study extends these findings highlighting the importance of the social environment within sporting organisations.

The findings of this study also suggest a disjunction between the focus of health and sport professionals who tend to focus on organisational/management issues such as alcohol management and the adolescent girls who focus on interpersonal issues. For instance, a panel of health and sport professionals were surveyed regarding 21 potential standards for health-promoting sports clubs related to seven health-promoting themes (i.e. healthy eating, sponsorship and fundraising, alcohol management, smoke-free environments, sun protection and social inclusion) [24]. They identified standards related to alcohol management such as responsible alcohol practices and restricting alcohol during junior sporting activities as the highest priority issue in terms of health promotion in sports clubs [24]. In contrast, only around a third of adolescent girls in the present study reported that sun protection and responsible alcohol practices were important; although the importance of responsible serving of alcohol trended upward over time for the older cohort (Year 11) and was significantly more important in non-metropolitan than metropolitan regions as previously discussed. However, some of the difference between the professionals and adolescent girls may be attributable to differences in the procedures and tools used in the two studies to measure HWE factors, which make comparisons difficult. Further, factors such as seasonality of sports, indoor vs outdoor sports participation and the liquor license status of sports clubs may also affect the perception of the importance of Sunsmart and alcohol practices.

Improving welcoming club practices might help to address participation barriers commonly reported by adolescent girls such as those associated with feeling self-conscious, being worried about body image or skill levels, and social support from peers and adults [35]. It can be very daunting for a young person to enter a sports club, with many adolescent girls reporting that they are worried about their appearance and performance and that they feel apprehensive about whether they will be accepted by others in the sports club [36]. Social support through family and friends [33, 34, 37], and the club itself as shown in the current study, is an important determinant for adolescent females’ participation in sport and physical activity. Adolescent girls have also perceived sports clubs as being exclusive or elitist [36]. Other researchers have highlighted problems of social exclusion and marginality within sport, particularly in rural Australia [38]. Researchers [36] have suggested that entry into and continued inclusion within sports clubs may not be the egalitarian process often espoused [39, 40]. Therefore, welcoming club practices may be important from the perspective of engaging and maintaining sports participation. The social environment of sport clubs could be assessed and evaluated to ensure strategies to ‘welcome’ and include adolescent girls are implemented. For example, strategies might involve a designated club ambassador who is responsible to meet and greet new members, run induction sessions to provide information on membership options, club events, club policies and practices, and link new members with a peer mentor. It might also be important to promote youth-friendly messages and communication strategies (e.g. social media) by clubs and coaches to create a welcoming environment and facilitate positive club-coach-participant-peer relationships.

The importance of human interactions and specifically club-coach-participant-peer relationships in sports clubs cannot be underestimated, especially to engage and retain adolescent girls through welcoming club practices. Research has shown that coaches have an important role for sustaining athlete involvement by creating a motivationally conducive atmosphere [41]. Ego-involving climates created by coaches (e.g. the coach pays most attention to the best players) and peers (e.g. look pleased when they do better than their teammates) can contribute to the formation of antisocial attitudes such as acceptance of gamesmanship, in contrast to task-involving climates (e.g. the coach says that all of us are important to the team’s success) and peers (e.g. encourage their teammates to try their hardest) [41]. Coté et al. [42] stated that coaches should “nurture intrinsic values of sport participation while endorsing and cultivating the social side of sport” (p.11), particularly for adolescents who are focused on participating recreationally in sport. It has also been suggested that coaches require education to capitalise on the opportunity they have to promote PA through organised sport [43].

A major strength of this study was the longitudinal design which examined the perceived influence of HWEs on participation in sports club among adolescent girls and how these influences changed over a 3 year period. However, we acknowledge some limitations to the study which have implications for interpretation of the results. First, the response rates were low, which was attributable at least in part to the ethical requirements of Australian education authorities to obtain specific ‘opt-in’ parental consent, exacerbated by the necessity to communicate with parents only indirectly in writing via the school and the students themselves. Second, there may have been self-selection bias, with more physically active girls being more motivated to participate in the study, which is difficult to avoid. Third, there may be other factors within the organisational environment and/or at the program level that influence sport participation among girls. For instance, one group of researchers from Sweden have reported that the reasons adolescent girls continue participating in sports clubs is that they are fun and provide a sense of belonging and improving sport skills, rather than the desire to compete [44, 45]. Future research could explore the influence of program-level factors like training demand, competition focus, and skill development, on sport club participation. Finally, three quarters of the sample were from the younger cohort, hence the study had less statistical power to detect differences and trends among the older adolescents.

Finally, the findings of this study are important for both sport and health-based organisations in attempts to engage adolescent girls in structured sport and/or physical activities for individual and community wellbeing. As highlighted by Misener and Misener [46] “health-related outcomes are entwined in both health and sport policy agendas” (p.3) and as a result there is an increasing emphasis placed on sport providers to collaborate with other sectors–including health and also education. For instance, in Australia, sport government departments are currently positioned within the Department of Health at state and national levels and aim to “increase participation in physical and recreational activities to promote physical and mental health” [47]. It is important emphasise that an integrated and coordinated approach to collaborative partnerships is required in the integration of sport and health promotion; and in particular one that addresses the limited capacity of community sports organisations [46, 48, 49].

## Conclusion

Healthy and welcoming sport club settings provide opportunities to encourage healthy behaviour and might influence club sport participation. This study found that adoelscent girls perceived the welcoming environment, along with two of the healthy environment practices (smoke-free environments and injury prevention strategies) as being positively influential in their decision to participate in a sports club. As such, these health promotion areas should be a priority for encouraging sport club participation among adolescent girls. In particular, the welcoming environment such as the friendliness of people within the club, knowing someone at a club and friendliness of the coach were influential factors for adolescent girls in sports club settings. Strategies that support the social environment within sports clubs need to be prioritised by sporting bodies and coaches to encourage participation in sport by adoelscent girls. Further, it is important to focus on specific health issues by age and region with responsible serving of alcohol being more important among older adoelscents and in non-metropolitan regions. Sport governing bodies could play an important role in designing and facilitating the implementation of such strategies, along with integrated and coordinated partnerships with health promotion organisations.

## Abbreviations

BMI: Body mass index
HWE: Healthy and welcoming environment
PA: Physical activity
PE: Physical education
SES: Socioeconomic status
